# Zoonotic vaccinia virus strains belonging to different genetic clades exhibit immunomodulation abilities that are proportional to their virulence

**DOI:** 10.1186/s12985-021-01595-z

**Published:** 2021-06-09

**Authors:** Karine Lima Lourenço, Leandro Andrade Chinália, Lethícia Ribeiro Henriques, Rodrigo Araújo Lima Rodrigues, Flávio Guimarães da Fonseca

**Affiliations:** 1 Laboratory of Basic and Applied Virology, Department of Microbiology, Institute of Biological Sciences, Federal University of Minas Gerais, Belo Horizonte, MG Brazil; 2 Technical Support Center for Teaching, Research and Extension, Institute of Environmental, Chemical and Pharmaceutical Sciences, Federal University of São Paulo, Diadema, SP Brazil; 3 Laboratory of Biology and Technology of Microorganisms, Department of Biological Sciences, Federal University of Ouro Preto, Ouro Preto, MG Brazil; 4 Laboratory of Viruses, Department of Microbiology, Institute of Biological Sciences, Federal University of Minas Gerais, Belo Horizonte, MG Brazil

**Keywords:** Poxvirus, Vaccinia virus, Immune response, Immune downmodulation, Guarani P1 virus, Passatempo virus

## Abstract

**Background:**

The vaccinia virus (VACV) isolates*,* Guarani P1 virus (GP1V) and Passatempo virus (PSTV), were isolated during zoonotic outbreaks in Brazil. Each one of them belongs to two different VACV clades, defined by biological aspects that include virulence in mice and phylogenetic analysis. Considering that information about how vaccinia viruses from different groups elicit immune responses in animals is scarce, we investigated such responses in mice infected either by GP1V (group 2) or PSTV (group 1), using VACV Western Reserve strain (VACV-WR) as control.

**Methods:**

The severity of the infections was evaluated in BALB/c mice considering diverse clinical signs and defined scores, and the immune responses triggered by GP1V and PSTV infections were analysed by immune cell phenotyping and intra-cytoplasmic cytokines detection.

**Results:**

We detected a reduction in total lymphocytes (CD3 +), macrophages (CD14 +), and NK cells (CD3-CD49 +) in animals infected with VACV-WR or GP1V. The VACV-WR and GP1V viruses, belonging to the most virulent group in a murine model, were able to down-modulate the cell immune responses upon mice infection. In contrast, PSTV, a virus considered less virulent in a murine model, showed little ability to down-modulate the mice immune responses. Mice infected with VACV-WR and GP1V viruses presented significant weight loss and developed lesions in their spleens, as well as damage to liver and lungs whereas mice infected with PSTV developed only moderate clinical signs.

**Conclusions:**

Our results suggest that VACV immunomodulation in vivo is clade-related and is proportional to the strain’s virulence upon infection. Our data corroborate the classification of the different Brazilian VACV isolates into clades 1 and 2, taking into account not only phylogenetic criteria, but also clinical and immunological data.

**Supplementary Information:**

The online version contains supplementary material available at 10.1186/s12985-021-01595-z.

## Background

**Fig. 1:**
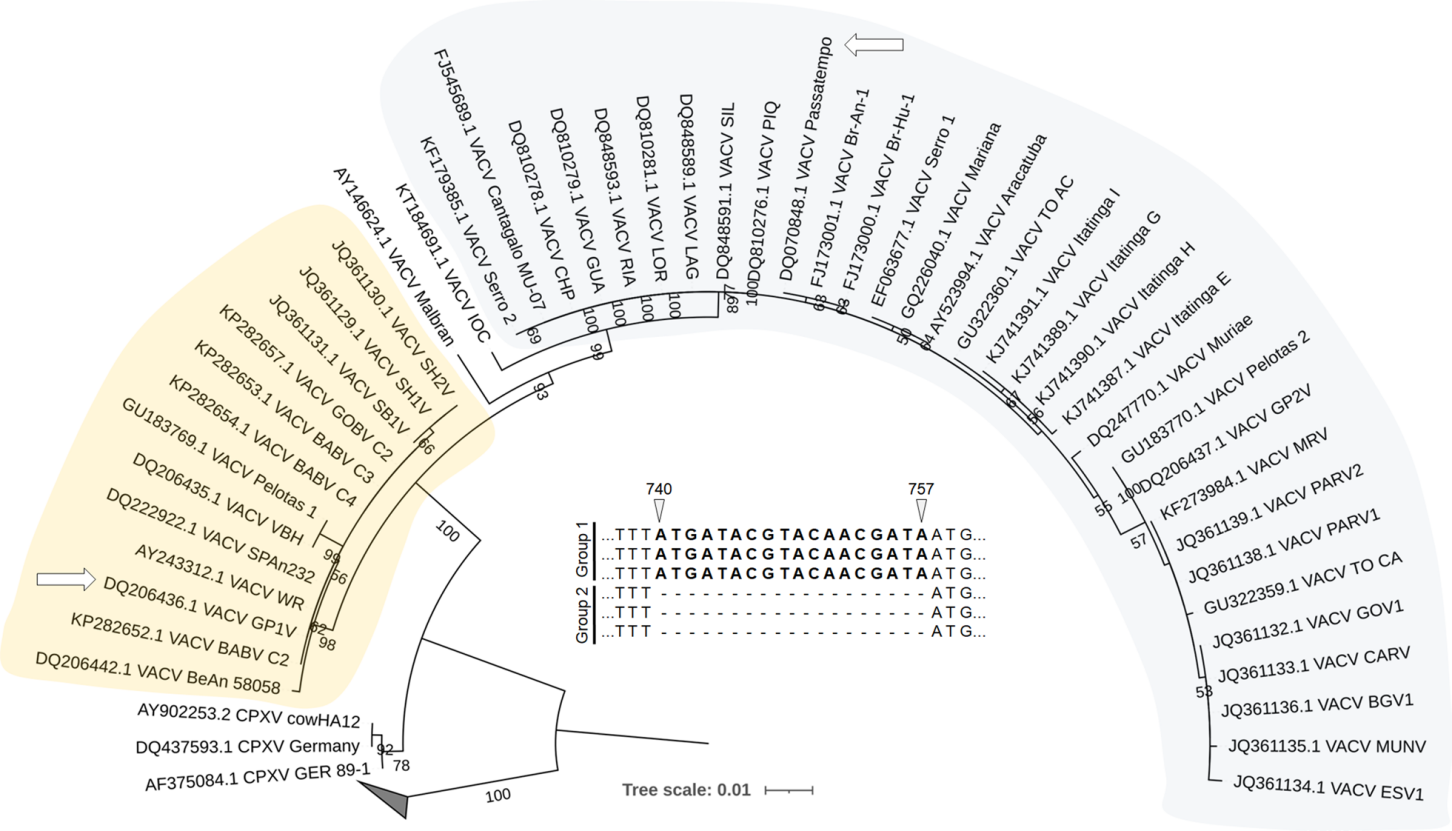
Phylogenetic analysis of VACV-BR based on nucleotide sequences of the A56R gene (hemagglutinin). VACV-BR group 1 is highlighted with light yellow and group 2 with light blue. White arrows indicate the Brazilian viruses used in this study. The alignment was built using Muscle with standard parameters [45]. The maximum likelihood tree was built with IQ-TREE [46]. The best-fit replacement model selected by ModelFinder was HKY + F + I. The branch supports were estimated using 1000 replicates of standard bootstrap and the tree was visualized with iTOL [47]. Bootstrap values lower than 50 were suppressed. The clade of variola viruses has been collapsed. The scale bar indicates the number of substitutions per site. A schematic alignment was included (inset) to indicate the genomic signature that divides VACV-BR into two distinct groups, an 18-nt deletion between positions 740 and 757 of the A56R gene. The representation was based on the VACV-WR (AY243312) gene

Members of the *Poxviridae* family present a double-stranded DNA genome that varies from 140 to 300 kbp in size, depending on the virus species. Their large genomes encode more than 150 genes, including some involved in immunomodulatory mechanisms [1–3]. In Brazil, vaccinia virus (VACV) has been circulating in rural and wild environments for decades[4]. Brazilian VACV isolates are currently classified into two groups: group 1 (less virulent in a murine model of infection), and group 2 (more virulent in a murine model of infection) (Fig. 1). In addition to virulence in mice, this division reflects biological properties of the isolates such as plaque phenotype in BSC-40 cells and genetic differences, including the presence or absence of an 18 nucleotide deletion in the viral hemagglutinin protein (HA A56R) coding gene (see inset on Fig. 1) [5].

The Guarani P1 virus (GP1V) and Passatempo virus (PSTV), used in this study, were isolated in Minas Gerais, Brazil, in 2001 and 2003 respectively, from outbreaks in rural properties involving cattle and humans [6, 7]. The infections by these viruses caused lesions on the udder, teats, snout and oral mucosa of cows and calves. Milkers usually develop hand lesions after unprotected contact with cows infected with these viruses and clinical signs such as high fever, severe headache, back pain and lymphadenopathy are frequently reported by individuals handling infected cows [7, 8].

Much of the replicative success of poxviruses is related to their capacity to obstruct, escape or subvert essential elements of their host's antiviral responses. It has been proposed that the poxviruses' ability to down-modulate the host's immune response is directly proportional to their virulence in vivo [9]. Previous studies have already shown a difference in the modulation of the immune response in mice infected with non-replicating (Modified vaccinia virus Ankara), attenuated (vaccinia virus Lister), and replicative (vaccinia virus Western Reserve) virus strains [9, 10].

The evaluation of infections caused by naturally circulating VACV strains GP1V (group 2) and PSTV (group 1) represents an opportunity to analyse immune responses triggered in the host as a result of infections with viruses presenting different virulence patterns. Such studies can, then, be compared to those including either laboratory strains of VACVs or vaccine strains. Here we investigated the immune responses triggered in BALB/c mice infected with the GP1V or PSTV VACV strains, originated from zoonotic outbreaks in Brazil, compared to infections by a laboratory reference strain, vaccinia virus Western Reserve (VACV-WR). Our results are important not only to better define patterns of immunomodulation in vivo, caused by zoonotic vaccinia viruses, but they also corroborate the genetic classification of feral VACVs into two separate clades, which has been subject of criticism.

## Methods

### Viruses

Samples of GP1V and PSTV strains were kindly provided by Dr. Erna Kroon (Universidade Federal de Minas Gerais, Belo Horizonte, Brazil) and VACV-WR was gently provided by Dr. Bernard Moss (NIAID/NIH, Bethesda, EUA). The three viral samples were grown and titrated (PFU/ml) in BSC-40 cells through plaque assay and purified in sucrose cushions, as described before [11].

### Infection of mice and clinical signs

BALB/c mice used in this study were obtained from UFMG's central animal facility (Belo Horizonte, Brazil) and maintained in our experimental animal facility (Departamento de Microbiologia, Belo Horizonte, Brazil). Animals were kept into ventilated cages with food and water ad libitum. All in vivo procedures were approved by the Committee of Ethics for Animal Experimentation (CETEA) from UFMG, under permission 9/2019. Six-week-old male mice were separated into groups infected with GP1V, PSTV, VACV-WR or mock-infected (control). Animals were anaesthetized by intra-peritoneal injection of ketamine and xylazine (70 mg/kg and 12 mg/kg of body weight in phosphate-buffered saline [PBS], respectively). The intranasal (i.n.) route was used to inoculate 10 μL of PBS or 10 μL of purified viruses diluted in PBS on subgroups of five animals each (for clinical signs and weight loss evaluation) or subgroups of seven animals each (histopathological analysis, splenocyte preparation, immunophenotyping and detection of intracellular cytokines)*.*

In order to monitor infections from a clinical perspective, groups of five animals each were inspected daily, starting from the inoculation day. Inoculums of 10^4^, 10^5^ and 10^6^ PFU of PSTV, VACV-WR or GP1V were used to infect the animals whilst the control group was inoculated with sterile PBS. Weight loss and clinical signs were evaluated for 10 days and registered.

### Histopathological analysis

Seven days post inoculation of 10^6^ PFUs of GP1V, PSTV, VACV-WR or PBS, animals were euthanized and had spleens, lungs and livers harvested for histopathological analysis. Fragments of organs were fixed with formalin for 24 h and dehydrated with increasing concentrations (from 70 to 100%) of ethanol. Tissue fragments were diaphanized in xylol and embedded in paraffin. The segments were sectioned in a microtome (5 μm) and stained using Hematoxylin and Eosin. A pathological characterization of these slide preparations was performed, through the attribution of clinical scores, considering the presence and distribution of inflammation, edema, pulmonary hemorrhage and inflammation along with hepatic and splenic reactive degeneration [12–14].

### Splenocyte preparation, immunophenotyping and detection of intracellular cytokines

In order to evaluate the production of interferon-γ (IFN-γ) and tumor necrosis factor α (TNF-α) by CD4 + and CD8 + T lymphocytes, mice were inoculated with 10^6^ PFUs of PSTV, GP1V or VACV-WR through the intranasal route. Their spleens were harvested 7 days post infection and the splenocytes were obtained after tissue maceration. For erythrocyte lysis, cell extracts were resuspended in deionized water and incubated on ice. PBS 10X was used to stop the lysis process. Cell proliferation assays were performed through splenocyte labelling with Bromodeoxyuridine (BrdU). 96-well plates containing 2 × 10^5^ spleen cells per well received stimulus of 10^4^ PFU of purified, UV-inactivated VACV-WR; concanavalin A (ConA); or just RPMI medium (Gibco, Carlsbad, USA). Plates were incubated at 37 °C in a 5% CO_2_ atmosphere for 72 h. The evaluation of cell proliferation was performed according to the BrdU Cell Proliferation Assay kit (Millipore, USA) manufacturer's instructions. For cell immunophenotyping, 96-well plates containing 2 × 10^5^ splenocytes per well were cultivated and incubated for 30 min at 4 °C, in the dark, with monoclonal antibodies. Cell surface markers CD4, CD8, CD19, CD3/CD49 and CD14, as well as the costimulatory molecules CD25, CD28, CD80 and CD86 were evaluated. Plates were washed with PBS, centrifuged and cells were suspended in 200 μL of Macs Facs Fix (MFF) fixative solution.

For the purpose of detecting intracellular cytokines, 10^7^ cells extracted from the macerated spleens were stimulated overnight with UV-inactivated VACV-WR (10^4^ PFU) and incubated for 4 h at 37 °C with Brefeldin A (Sigma, MO, USA) at 1 mg/ml. Cells were washed in FACS buffer and stained with anti-CD4 and anti-CD8 antibodies (BD Pharmingen, NJ, USA) for 30 min at 4 °C in the absence of light. Cells were permeabilized with FACS buffer containing 0.5% saponin, and then stained with mouse anti-TNF-α, anti-IFN-γ, anti-Interleukin 4 and anti-Interleukin 10 (BD Pharmingen, NJ, USA) for further 30 min at room temperature. A new washing step with FACS buffer containing 0.5% saponin was followed by two washes with FACS buffer only. Cell preparations were stored at 4 °C in the dark after fixation using FACS fix solution. A FACSCalibur cytometer (Becton, Dickinson, NJ, USA) was used for flow cytometry, and further analyses were performed using FlowJo software—parameters granularity (SSC) versus size (FSC) (TreeStar Inc., OR, USA). The gating strategy for the flow cytometry analyses are presented on Additional file 1: figure S 1.

### Statistical analysis

The data was compared by analysis of variance (ANOVA) using Tukey post-test and parametric Student's *t* test. *p* values under 0.05 were considered significantly different. Statistical analyses were performed using Prism 8 software (GraphPad Software).

## Results

### Clinical signs in infected mice

**Fig. 2:**
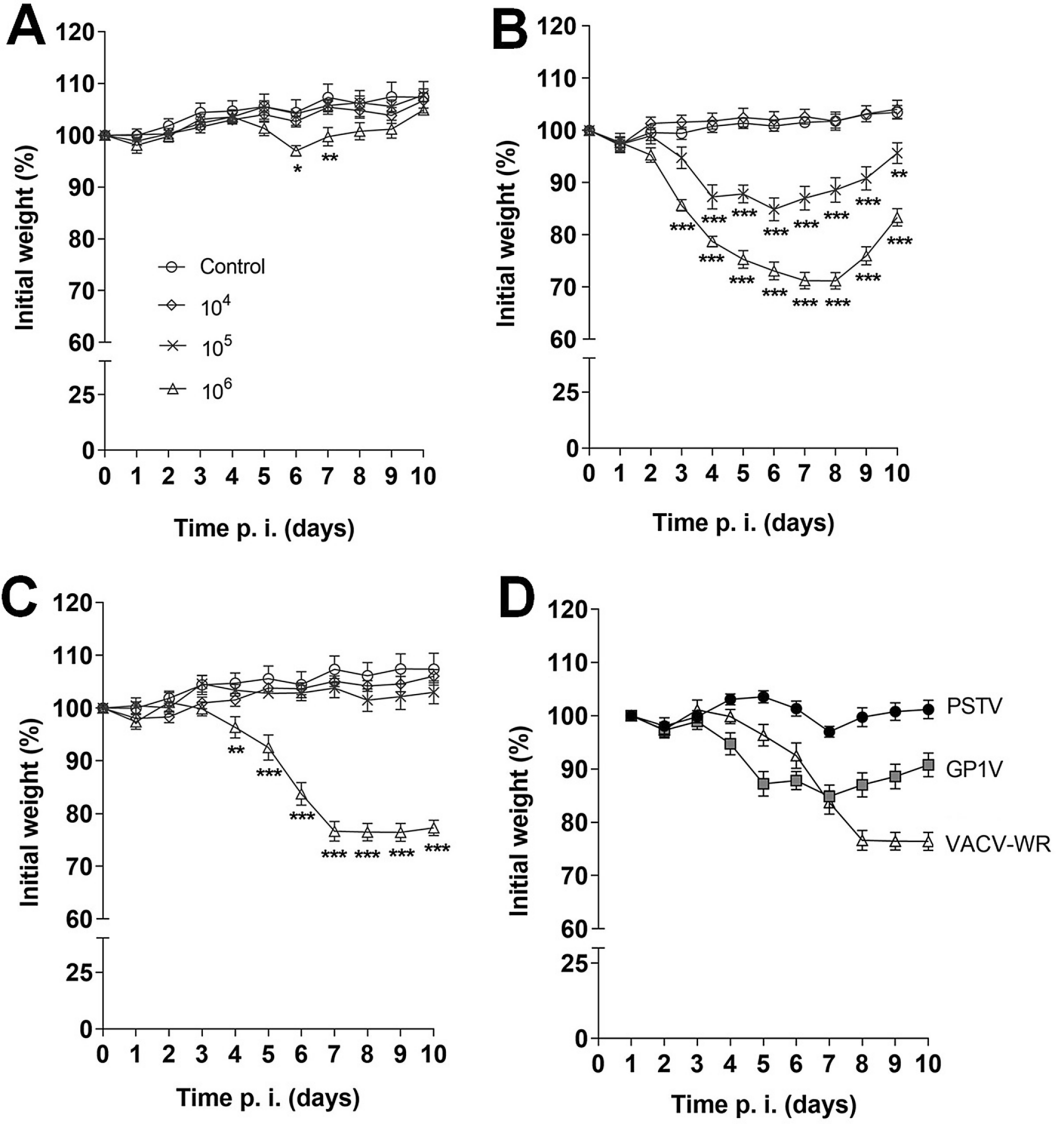
Body weight of BALB/c mice uninfected or infected with VACV-BR. Groups of mice (n = 5) were inoculated with PBS or 10^4^, 10^5^, 10^6^ PFU of PSTV (**A**), GP1V (**B**) or VACV-WR (**C**) by the intranasal route. Panel **D** represents the comparative weight loss in animals infected with 10^6^ PFU of each one of the three viral samples. The mice’s weight was determined daily and those that lost more than 30% of their initial weight were euthanized. The percentages of median weight relative to their initial weight were plotted. The error bars indicate the standard deviations. Significance variations were calculated using the Two-way ANOVA post hoc Bonferroni with statistical significance: *p < 0.05, **p < 0.01, ***p < 0.001

Animals infected with PSTV did not show typical clinical signs of infection by virulent strains of VACV, which include piloerection, important weight loss, arched back, swelling of the face, and conjunctivitis. Infection with 10^6^ PFU of PSTV led to a slight weight loss on days 6 and 7 post-infection, but animals soon recovered (Fig. 2A). All the mentioned signs were observed in animals infected with GP1V and VACV-WR and their severity were dose-dependent. Animals infected with 10^6^ PFUs of GP1V lost up to 28.84% of their initial weight (Fig. 2B) and weight loss in animals infected with 10^6^ PFUs of VACV-WR was close to 30% (Fig. 2C). Mice infected with VACV-WR developed more severe clinical signs when compared to animals infected with the same inoculum of GP1V. These results were similar to data presented on a previous study; such replication was necessary in order to set a baseline for all further experiments and evaluations.

### Histopathological analysis of liver, spleen and lung

**Fig. 3:**
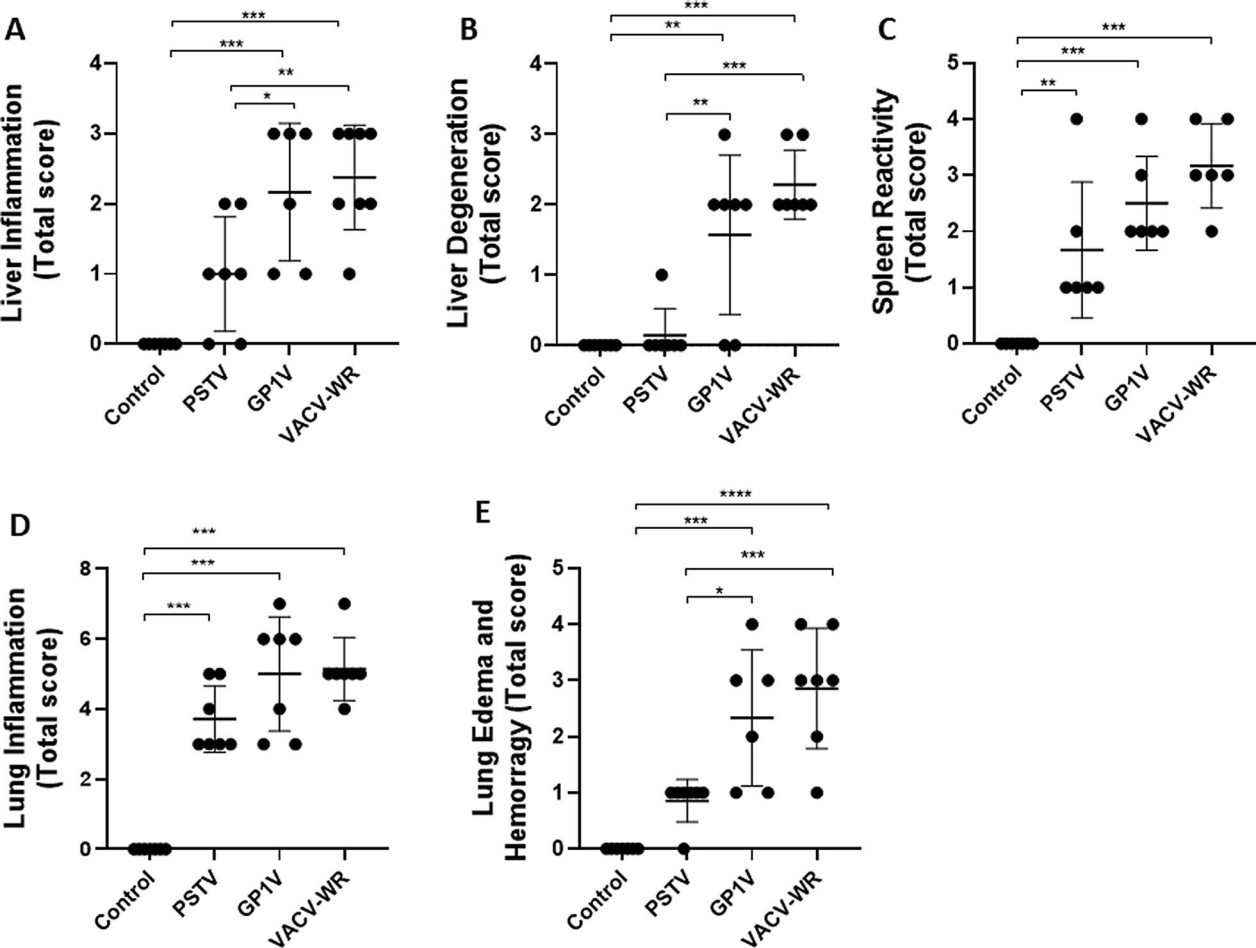
Pathological analysis of liver, spleen and lung of BALB/c mice uninfected or infected with VACV-BR. Groups of mice (n = 7) were inoculated with PBS or 10^6^ PFU of PSTV, GP1V or VACV-WR by the intranasal route. The liver inflammation (**A**) and degeneration (**B**), spleen reactivity (**C**), lung inflammation (**D**), edema and hemorrhage (**E**) were determined 7 days post infection. Scores were assigned to severity and distribution of the assessed characteristic on the tissue and these values were converted to a total score. For severity and distribution, the scale adopted scores from 0 to 5 or 0 to 4, in which the maximum value corresponds to a greater severity and distribution of the pathological alteration in the studied tissue. The pathological scores were plotted. The error bars indicate the standard deviations. Significance variations were calculated using the One-way ANOVA post hoc Bonferroni with statistical significance: *p < 0.05, **p < 0.01, ***p < 0.001

The pathological characterization of the lungs, livers and spleens of mice uninfected or infected with 10^6^ PFUs of GP1V, PSTV or VACV-WR was performed through the attribution of clinical scores. Scores were given in relation to severity and distribution of the evaluated parameter on the tissue and these values were converted to a total score. For severity and distribution, the scale adopted scores from 0 to 5 or 0 to 4, in which the maximum value corresponds to a greater severity and distribution of the pathological alteration in the studied tissue. Animals infected with GP1V or VACV-WR developed more intense liver inflammation and degeneration when compared to animals of the control group and to those infected with PSTV (Fig. 3). All infected mice showed higher levels of splenic reactivity (hyperplasia of the splenic white pulp) and pulmonary inflammation when compared to mock-infected animals. Greater levels of oedema and pulmonary haemorrhage were found in the GP1V- and VACV-WR-infected animals compared to the uninfected controls and PSTV-infected animals. The histological findings are consistent with the observed clinical signs.

### Splenocyte proliferation after VACV stimulation and characterization of immune cells subpopulations elicited during VACV infection

**Fig. 4:**
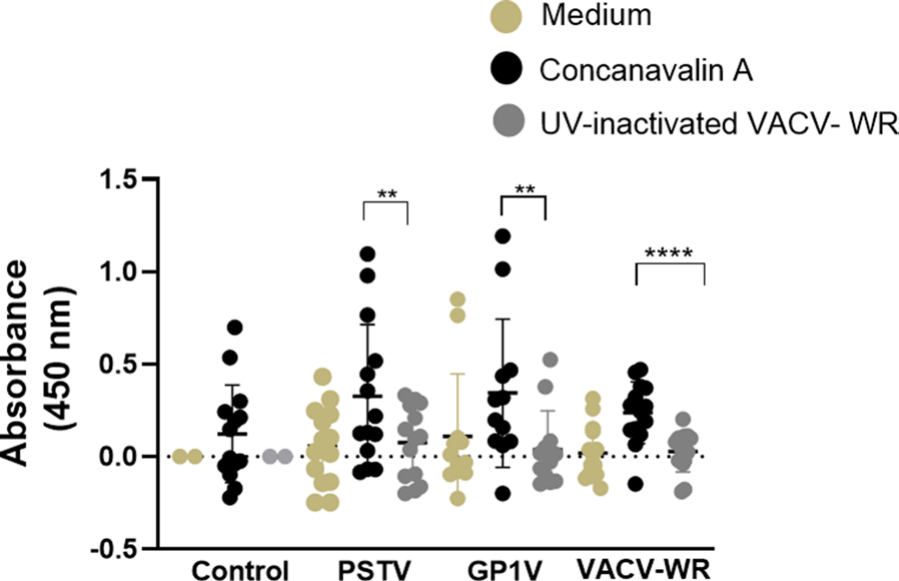
Proliferation of splenocytes from BALB/c mice uninfected or infected with VACV-BR. Groups of mice (n = 7) were inoculated with PBS or 10^6^ PFU of PSTV, GP1V or VACV-WR by the intranasal route. The spleen cells proliferation was determined 7 days post infection with BrdU Cell Proliferation Assay (Millipore, EUA) by measuring the absorbance at 450 nm. For that, spleen cells were cultured in the presence of 2 µg/mL of Concanavalin A (black dots), 10^4^ PFU of UV-inactivated VACV-WR (gray dots) or mock-treated with medium (light brown dots). The absorbance values were plotted. The error bars indicate the standard deviations. Significance variations were calculated using the One-way ANOVA post hoc Bonferroni with statistical significance: *p < 0.05, **p < 0.01, ***p < 0.001, ****p < 0.0001. During the experiment some animals were lost (The local Committee of Ethics for Animal Experimentation does not allow the replacement of animals to compensate for this loss)

Proliferation rates of spleen cells were higher in samples from infected mice stimulated with ConA compared to those stimulated with the UV-inactivated VACV-WR (Fig. 4). This increase was similar for splenocytes from GP1V- and PSTV- infected animals and less pronounced for VACV-WR infected animals.

**Fig. 5:**
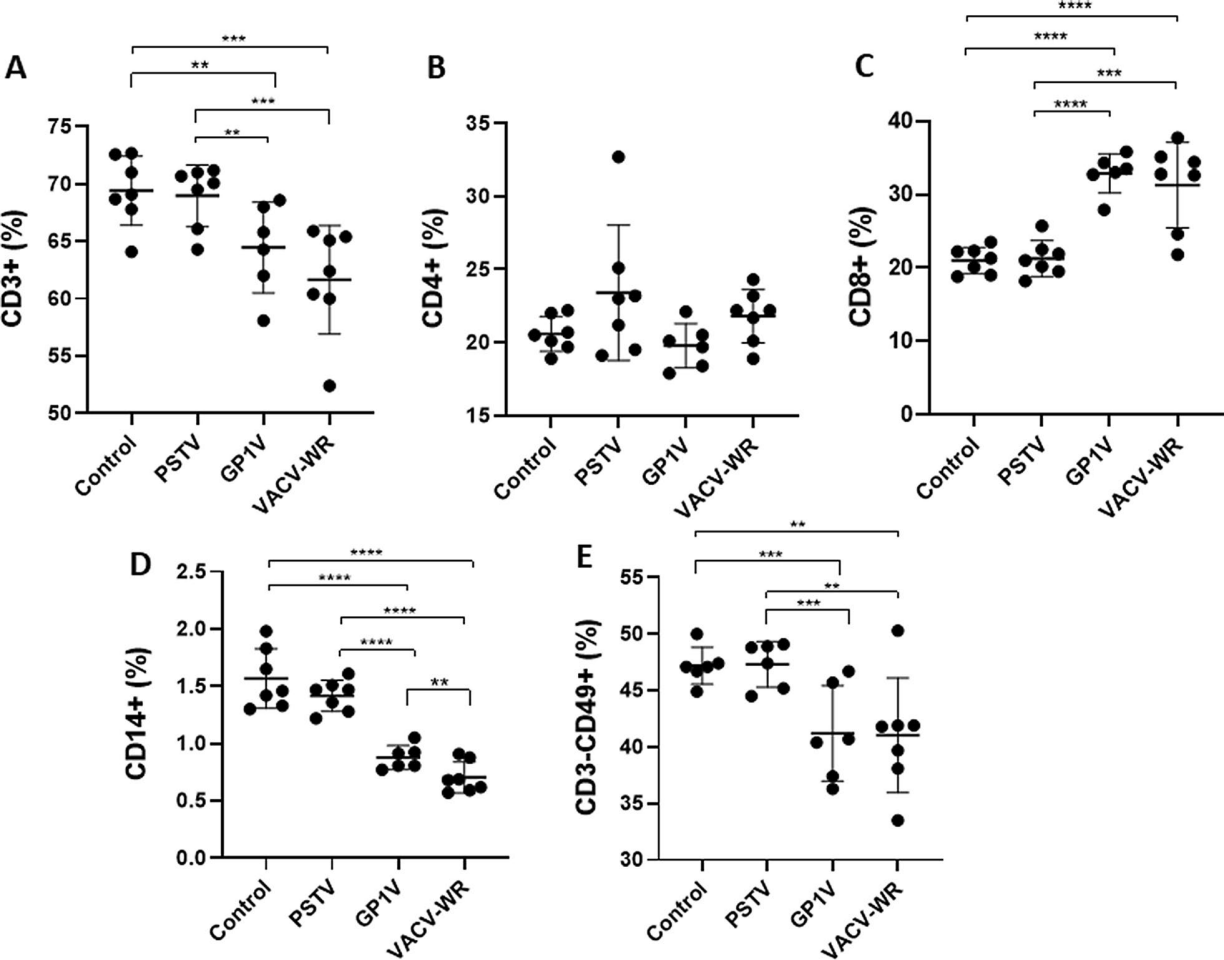
Immunologic populations from spleen of BALB/c mice uninfected or infected with VACV-BR. Groups of mice (n = 7) were inoculated with PBS or 10^6^ PFU of PSTV, GP1V or VACV-WR by the intranasal route. The percentage of T lymphocytes CD3 + (**A**), CD4 + (**B**) and CD8 + (**C**), monocytes CD14 + (**D**) and NK cells CD3-CD49 + (**E**) was determined 7 days post infection by flow cytometry and analyzed using FlowJo software, parameters granularity (SSC) versus size (FSC). The error bars indicate the standard deviations. During the experiment some animals were lost. CETEA does not allow us to increase the number of animals to compensate for this loss. Significance variations were calculated using the One-way ANOVA post hoc Bonferroni with statistical significance: *p < 0.05, **p < 0.01, ***p < 0.001, ****p < 0.0001

Analyses of subpopulations of splenic immune cells, including T-helper cells, B lymphocytes, NK cells and monocytes from uninfected mice or animals infected with 10^6^ PFUs of GP1V, PSTV or VACV-WR were performed (Fig. 5). A reduction in the frequency of CD3 + cells was observed in the animals from the group infected with VACV-WR and GP1V in comparison to those from the groups infected with PSTV or uninfected (Fig. 5A). A higher statistical significance was found when PSTV-infected or uninfected animals were compared to VACV-WR than when compared to GP1V. Although no differences were detected in the frequency of CD4 + cells in the evaluated groups (Fig. 5B), CD8 + cells were more frequent in groups infected with GP1V and VACV-WR, respectively, in comparison to those infected with PSTV or uninfected controls (Fig. 5C). There was a decrease in the frequency of CD14 + and CD3-CD49 + cells in the groups infected with VACV-WR and GP1V when compared to the PSTV and the uninfected control group (Fig. 5D-E). CD14 + subpopulation was even less frequent in animals infected with VACV-WR than in animals infected with GP1V. The unique ability of VACV strains to down-modulate subsets of the host immune cells has been described [10], including for human infections by zoonotic samples of Brazilian VACV [9].

**Fig. 6:**
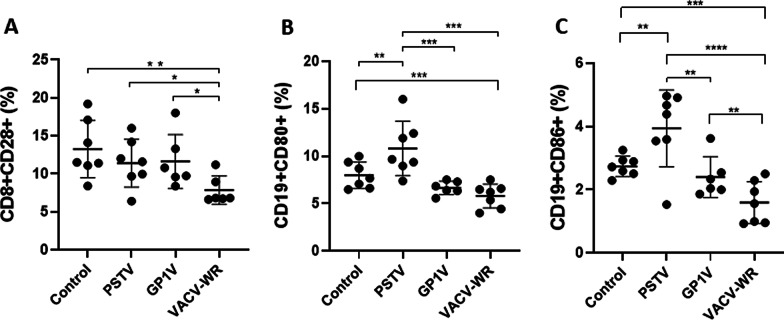
Activated phenotype in CD8 + T-cell and B lymphocyte from BALB/c mice uninfected or infected with VACV-BR. Groups of mice (n = 7) were inoculated with PBS or 10^6^ PFU of PSTV, GP1V or VACV-WR by the intranasal route. The mean fluorescence intensity of CD8 T lymphocytes expressing CD28 (**A**) surface activation marker and percentage of B lymphocytes CD19 expressing CD80 (**B**) or CD86 (**C**) was determined 7 days post infection. The cells stained with monoclonal antibodies linked to fluorochromes against cell surface molecules were detected by flow cytometry. The error bars indicate the standard deviations. Significance variations were calculated using the One-way ANOVA post hoc Bonferroni with statistical significance: *p < 0.05, **p < 0.01, ***p < 0.001, ****p < 0.0001. During the experiment some animals were lost (The local Committee of Ethics for Animal Experimentation does not allow the replacement of animals to compensate for this loss)

The activation profile of CD8 + T cells, observed through the analysis of the expression of the CD28 + molecule, suggested that only animals infected with VACV-WR had a significant reduction in the activation profile of these cells (Fig. 6A). The modulation of CD8 + CD28 + cells after infection by the VACV-WR virus in mice has been demonstrated previously, as opposed to infections by VACV Lister and *modified Vaccinia virus Ankara* strains [9]. By analysing B lymphocytes expressing CD80 we observed that the frequency of these cells' subset decreased similarly in mice infected with VACV-WR and GP1V in comparison to animals inoculated with PSTV (Fig. 6B). On the other hand, animals infected with PSTV presented more CD80 + B cells than uninfected control animals (Fig. 6B). Compared to the control and PSTV groups, the frequency of CD19 + CD86 + cells in animals infected with VACV-WR was lower. Similarly, GP1V group showed a significant reduction in these cells compared to PSTV group (Fig. 6C). This reduction in the frequency of CD80 + and CD86 + B lymphocytes was also reported for individuals affected by bovine vaccinia in Brazil [10].

### Production of lymphocytic cytokines during infection by different VACV

**Fig. 7:**
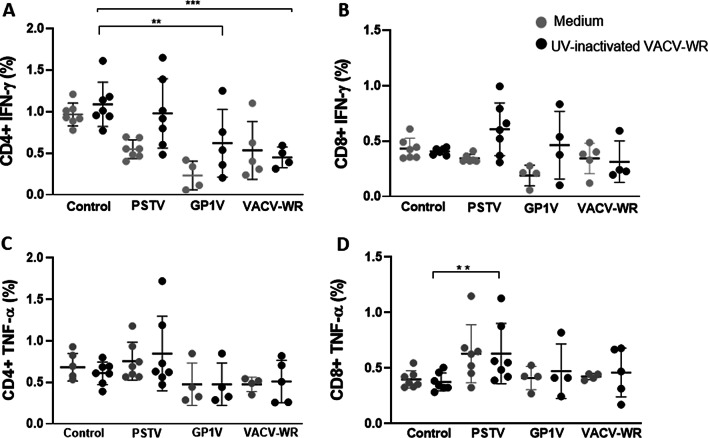
Interferon-gamma and tumor necrosis factor production from BALB/c mice uninfected or infected with VACV-BR. Groups of mice (n = 7) were inoculated with PBS or 10^6^ PFU of PSTV, GP1V or VACV-WR by the intranasal route. The percentage of T lymphocytes CD4 + expressing IFN-γ (**A**) and TNF-α (**C**) or CD8 + expressing IFN-γ (**B**) and TNF-α (**D**) was determined 7 days post infection by flow cytometry. For that, spleen cells were cultured in the presence of 10^4^ PFU of UV-inactivated VACV-WR (black points) or mock-treated with medium (gray points) and were labeled with mouse anti-IFN-γ and anti-TNF-α antibodies. The error bars indicate the standard deviations. Significance variations were calculated using the One-way ANOVA post hoc Bonferroni with statistical significance: *p < 0.05, **p < 0.01, ***p < 0.001, ****p < 0.0001. During the experiment some animals were lost (The local Committee of Ethics for Animal Experimentation does not allow the replacement of animals to compensate for this loss)

We observed a general decrease in the IFN-γ-producing CD4 + T lymphocytes for the infected groups (PSTV, GP1V and VACV-WR) compared to the uninfected controls, in cells stimulated or not with UV-inactivated VACV-WR (Fig. 7A). The same trend was not observed when the IFN-γ-producing CD8 + T cells were analysed (Fig. 7B). The production of TNF-α by CD4 + T lymphocytes was similar for all groups evaluated after virus-antigen stimulation or no stimulation (Fig. 7C). Although the levels of TNF-α-producing CD8 + lymphocytes were slightly higher in animals infected with PSTV, compared to all other groups, (Fig. 7D) it was clear that the effect of VACV infection in TNF production by T cells is much more subtle than for IFN-γ production.

## Discussion

The importance of innate, cellular and humoral immunity components on fighting orthopoxvirus' infections has been demonstrated in several studies. The depletion of macrophages in mice results in their inability to control infection by Vaccinia virus [15]. Likewise, the decline of NK cells levels in C57BL/6 mice culminates in increased ectromelia virus (ECTV) titters and disease severity [16, 17]. Complement-deficient mice developed more severe disease when infected with cowpox virus [18]. Evaluation of cytokines, such as IFN (I and II) and TNF also confirmed the key role of these molecules in the innate immune response against orthopoxviruses [19–22]. Both cellular and humoral responses are highly coordinated and require the combined activity of B and T lymphocytes. The primary infection of mice by ECTV cannot be controlled exclusively by TCD8 + lymphocytes [23] and production of antibodies by B lymphocytes is also essential in disease control, reinforcing the functional complementarity of the immune responses upon poxvirus infections. This interaction between B and T cells is also crucial in subsequent exposures to these viruses [24]. Nonetheless, poxviruses encode several proteins that are related to the evasion of the host’s immune response [25, 26]. Indeed, it has been demonstrated that poxviruses infecting humans are able to significantly modulate components of host-specific immune response [27, 28]. Likewise, many studies have demonstrated the immunomodulatory ability of poxviruses in animal infections [9, 17, 26, 29]. Therefore, these viruses' ability to block, escape or subvert the essential elements of the antiviral response is essential for their replicative success in the host [25]. 

The Brazilian VACV isolates are currently divided into two distinct groups. This classification considers characteristics such as the virulence of these isolates in a murine model of infection, which in turn is linked to intrinsic genetic differences in their respective genomes [30]. Analyses on how different zoonotic VACV isolates interact with their hosts, as well as other virological and biological aspects, could reinforce and support their segregation and classification into different genetic groups.

In this study, we showed how VACVs that belong to genetically different groups are able to modulate mice immune responses in distinct patterns. Infections with VACV can result in clinical signs such as piloerection, weight loss, back arching, and facial oedema. Nonetheless, animals infected with different VACV isolates show these signs differently [9, 31]. Ferreira and colleagues have demonstrated that infection by VACV-WR and GP1V in mice led to the appearance of signs such as piloerection, back arching, periocular alopecia and 25% weight loss. In contrast, the same study showed that animals infected with PSTV and other VACV belonging to group 1, such as the Araçatuba virus and Guarani P2 virus samples, did not exhibit typical clinical signs of the infection and did not experience marked weight loss. We have replicated these experiments and observed that animals infected with GP1V and VACV-WR presented the typical signs of VACV infection belonging to group 2. On the other hand, mice infected with PSTV showed no obvious clinical signs after virus inoculation.

Poxviruses have an extensive capacity to infect different hosts. However, viral multiplication rates vary according to the host species, considering that it depends on host-specific antiviral mechanisms [32]. The acute infection initiated in the lung after VACV intranasal inoculation can spread to other organs of the host [31]. One hundred percent of the animals inoculated with the GP1V and VACV-WR showed chronic interstitial pneumonia, however, animals infected with PSTV showed only subtle signs of infection. Liver and spleen were also compromised by infection with viral samples, indicating that PSTV, GP1V and VACV-WR multiply initially in the lungs, spreading to other organs and causing systemic disease. We also found that only VACV-WR was able to cause pulmonary haemorrhage in animals. The histopathological evaluation of the samples showed that PSTV is associated with a lower degree of liver and splenic damage when compared to the other studied viruses (as shown on Fig. 3), similarly to what was described by Ferreira and collaborators [31].

As previously reported, both cellular and humoral immunities are important for controlling infections triggered by orthopoxviruses. Cell proliferation analysis is a parameter to detect the presence of antigen-specific lymphocytes, allowing us to obtain information about the cellular responses induced by the infection. Gomes and collaborators [10] performed cell proliferation experiments in human peripheral blood mononuclear cells (PBMCs) from individuals naturally infected with zoonotic VACV. They observed that after mitogenic and antigenic stimulation, individuals naturally infected with VACV showed a significant proliferative cell response compared to uninfected individuals. Similarly, our results showed increased levels of cell proliferation, after stimulation with VACV-WR, in cells from animals infected with the VACV-WR, GP1V and PSTV samples (Fig. 4).

To deceive the cellular and humoral immune responses, poxviruses encode several proteins capable of modulating their hosts' immune system. Gomes and collaborators [10] showed a lower frequency of CD14 + and an increase in CD8 + in humans infected with VACV zoonotic viruses. The immunomodulation of these subsets of cells suggests that such cells are important in controlling primary infection and preventing viral multiplication in infected cells. Furthermore, several studies have shown that the depletion of CD4 + T lymphocytes, macrophages and NK cells leads to greater disease severity in mice inoculated with VACV [17, 19, 33]. Overall, these viruses have developed specific down-modulation mechanisms for most immune cells that are important to counter the infection. Our data reflect the differences in patterns of immune responses triggered by different feral VACV strains and different abilities to down-modulate such responses, culminating in distinct patterns of virulence. Infections by the GP1V and VACV-WR viruses (VACV Group 2) resulted in a robust T CD8 + response, unlike the animals infected with the PSTV sample (VACV Group 1), which presented immune patterns that were similar to those observed in the mock-infected group. Indeed, CD8 + T cells play an essential role after infection by virulent VACV samples, but against less virulent VACV samples, CD8 + T cells are not so obviously required. [34, 35]. In addition, a reduction in total lymphocytes, NK cells and macrophages were observed in the group infected with VACV-WR. However, once again animals infected with the virus strain belonging to the phylogenetic group whose virulence characteristics in mice are milder or non-existent did not show variation in these cell subpopulations, presenting a global profile that was similar to the mock-infected animals. Cell activation patterns were also different when different VACV strains were inoculated in mice. VACV-WR- and GP1V-infected animals showed a tendency in CD19 + CD80 + cells down-modulation when compared to the uninfected controls. This was also observed in the study of VACV infections in humans [22]. Antibody production by B lymphocytes is essential to control infections caused by VACV [25]; therefore, it is not surprising that these viruses developed countermeasures that inhibit the activation of such cells. Mice infected with VACV-WR showed lower expression of CD28 in CD8 + T lymphocytes when compared to uninfected controls. Similarly, it has been suggested that this molecule is responsible for enhancing the activation of T cells after infection in mice with this VACV strain [9].

Cytokines are secreted, water-soluble proteins that act as mediators of immune responses, with autocrine and/or paracrine action. VACVs produce virokines and viroceptors that mimic the molecules of the host's immune system, mainly affecting IFN, TNF and other cytokines [36, 37]. IFN-γ has several important roles in the host's immune response, acting mainly in the regulation of the adaptive immune response and in the activation of macrophages [38]. Poxviruses encode proteins that are known to counteract IFN production or activity in their hosts [26]. Protein B18 has regions of homology with IFN-α/β receptor, and protein B8 is homologous to the IFN-γ receptor. These proteins prevent binding of IFN to cellular receptors, emphasizing the importance of this cytokine in the host's response to poxvirus infection [39, 40]. In addition, the products of the K3L and E3L genes, encoded by vaccinia virus, prevent the inhibition of IFN-induced protein synthesis [41, 42]. It is noteworthy that full genomic sequences for both Brazilian isolates used in this study are not yet available. Nevertheless we can expect these protein-coding genes are conserved in these viruses, considering the high degree of genetic conservation among members of *Orthopoxvirus* genus [43]. We observed a reduction in IFN-γ production by CD4 + T lymphocytes in animals infected with GP1V and VACV-WR after antigen stimulation (Fig. 7A). This cytokine participates in the activation of macrophages, stimulation of inflammation and the mounting of Th1-type responses, all essential for the control of viral infections [44]. The reduction of IFN-γ production by these cells in GP1V- and VACV-WR-infected mice emphasizes the immunomodulatory capacity of these viruses as opposed to the PSTV strain.

## Conclusions

Our data support a model in which the primary immune responses to acute orthopoxvirus’ infections have the involvement of Macrophages/Monocytes and possibly CD4 + T cells. The observation of modulation of those compartments in both humans [10] and mice, reinforces this hypothesis [12]. Finally, we demonstrated here that zoonotic vaccinia viruses belonging to different clades exhibit immunomodulation properties that are proportional to their pathogenic potential. These observations reinforce the idea that the segregation of zoonotic VACVs in two distinct clade/groups reflects not only genetic differences, but distinct virological and biological aspects as well.

## Supplementary Information

**Additional file 1: figure S 1**. Gating strategy for the flow cytometry analyses. Although not shown in the figure, analyses of the costimulatory molecules CD25, CD28 and CD86 were conducted using the same strategy.

## Data Availability

Data and materials are available upon request from the corresponding author.

## Abbreviations

BrdU: Bromodeoxyuridine
CETEA: Comitê de Ética em Experimentação Animal
ConA: Concanavalin A
ECTV: Ectromelia virus
FACS: Fluorescence-activated single cell sorting
FSC: Forward scatter
GP1V: Guarani P1 virus
IFN-γ: Interferon
IN: Intranasal
MFF: Macs facs fix
NK: Natural killer
PBMC: Peripheral blood mononuclear cell
PBS: Phosphate-buffered saline
PFU: Plaque-forming unit
PSTV: Passatempo virus
SSC: Side-scattered light
TNF: Tumor necrosis factor
UV: Ultraviolet
VACV: Vaccinia virus
VACV-WR: Vaccinia virus Western Reserve
VACV-BR: Brazilian Vaccinia viruses
