# Supplementary figures and images for: Zoonotic vaccinia virus strains belonging to different genetic clades exhibit immunomodulation abilities that are proportional to their virulence
Source: Virol J. 2021 Jun 9;18:124. doi: 10.1186/s12985-021-01595-z (PMC8191050; doi:10.1186/s12985-021-01595-z)

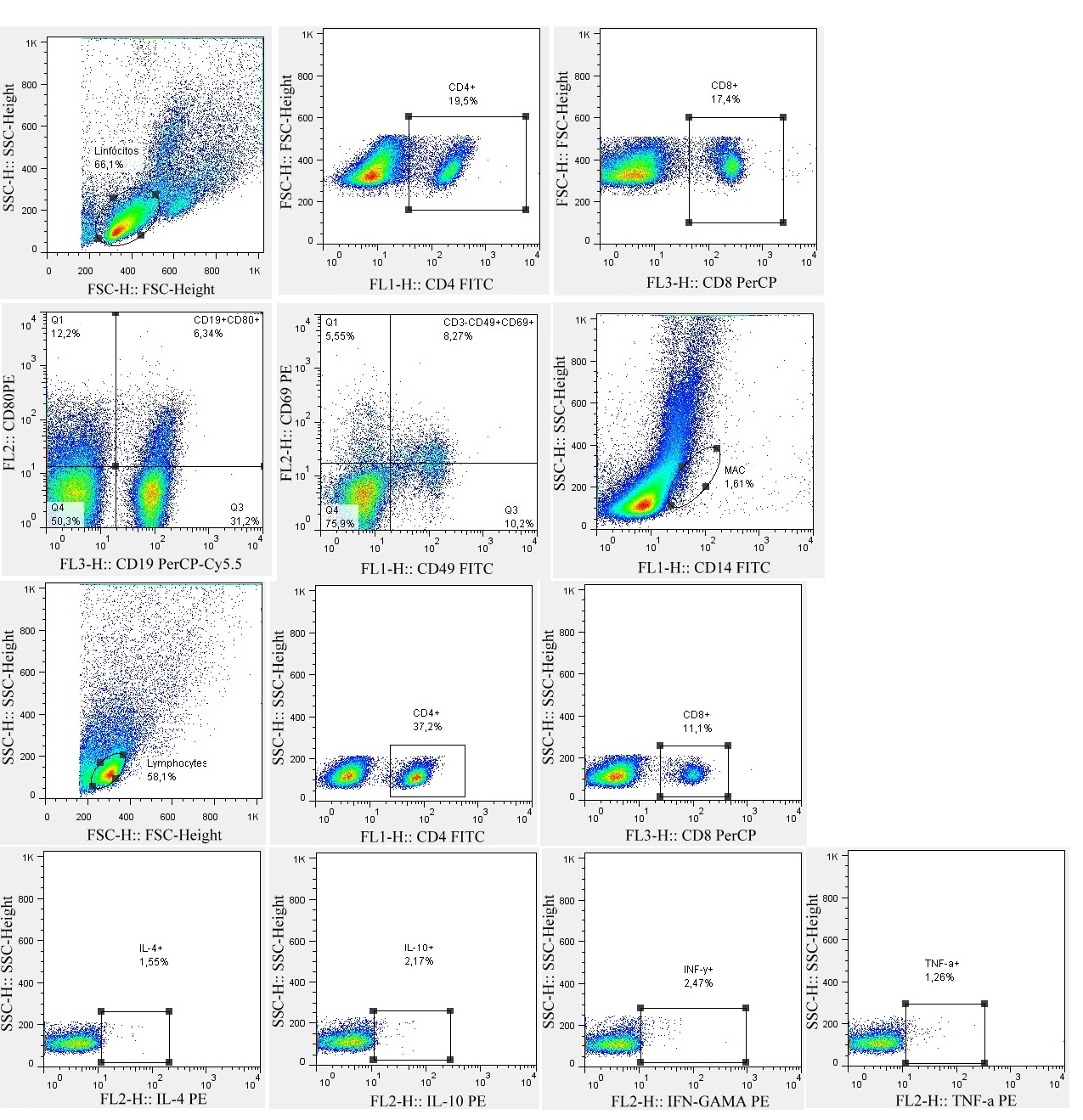

Supplement: Supplementary file 1 — Additional file 1: figure S 1. Gating strategy for the flow cytometry analyses. Although not shown in the figure, analyses of the costimulatory molecules CD25, CD28 and CD86 were conducted using the same strategy. [file 12985_2021_1595_MOESM1_ESM.tiff]
